# A Suggestion upon the Preparation of the Fingers and Nails for Surgical Operations

**Published:** 1894-08

**Authors:** Oscar H. Allis

**Affiliations:** Philadelphia.


ARTICLE III.

### A SUGGESTION UPON THE PREPARATION OF THE FINGERS AND NAILS FOR SURGICAL OPERATIONS.

OSCAR H. ALLIS, M. D., PHILADELPHIA.

Read before the Philadelphia County Medical Society, June, 1894.

The nails form no mean part of a surgeon's outfit. As a covering to the finger they give confidence; in the threading of needles they are often indispensable while often, when working among adhesions, they may serve a good turn. If the nails are too long they are in the way, and if too short, a privation. A medium length of nail is an exceedingly valuable helper at times. With some the length of nail is governed by the ability to keep it clean. Hence the nail is kept very short— much to the disadvantage of prehension, in which man excels.

The surgical care of the nails has had its full share of attention. The nail-brush forms a part of every physician's and surgeon's outfit. It is cheap, compact, and moderately thorough. Its disadvantages are that if stiff it is apt to scratch the hand or cut beneath the nails; if soft, it is of little value. To supplement the defects of the brush, some persist in using the point of the nail blade of their pocket-knifes. I say persist in using—as much has been written against the practice. Not only is there danger of cutting the flesh beneath the nail, but it leaves the under surface of the nail rough, making it a ready collector of filth, and less easily cleaned for a subsequent operation.

To avoid the knife I have long used a little wedge-shaped piece of soft pine. This, when wet, frays up, makes a kind of mop, is a good carier of soap, and enables me to wash out under the nail. The objection to my device was that the pine too rapidly frayed out, became bulky, and required frequent trimming. Finally I hit up-on the rubber eraser. A variety is made for artists and school children that is wedge shaped. This is ready for use as it is found at the stationer's, though if made a little sharper it is softer and more like a mop. It is pliable, soft and an excellent carrier of soap.

For the hand, generally the old-fashioned wash-rag cannot be improved upon. It is a good carrier of soap, and with it each finger in turn can be tightly caught and wrung until it is clean. With the nail or hand-brush only the back and front of the fingers get the scrubbing.

In addition to the implements usually deemed important for the cleanliness of the under surface of the nails, a very valuable one is the nail itself. Noticing that a young lady's fingers whom I frequently met were exceedingly neat, I made bold to ask her method, and was surprised to find that she had nothing more modern than a pair of scissors to trim her nails, and that with the wash-rag and the tips of her finger-nails she kept her hands in most perfect order. One thing that may be said of the finger nail as a nail-cleaning instrument, is that it will not scratch the under surface of the nail, a very important factor in the process, whether one aims at beauty or cleanliness.—*Medical and Surgical Reporter.*

